# Can we manage prophylactic therapy in COVID-19 patients to prevent severe illness complications?

**DOI:** 10.1590/1677-5449.200057

**Published:** 2020-05-20

**Authors:** Paulo Eduardo Ocke Reis, Marcos Cesar Braga Lima

**Affiliations:** 1 Universidade Federal Fluminense – UFF, Departamento de Cirurgia Geral e Especializada, Niterói, RJ, Brasil.

**Keywords:** COVID-19, coronavirus, anticoagulants, thrombosis, prevention, hospital mortality

## Abstract

Many patients with COVID-19 have thromboembolic complications that worsen their prognosis. Herein, the authors propose a modified version of the CHA_2_DS_2_-VASc score, including 1 point for COVID-19, so that prophylaxis to protect against thromboembolic events would be indicated before the condition becomes severe. The advantages of this modification would be prevention of the patient’s condition worsening due to thromboembolic problems and reduction of the likelihood of a need for intensive care and mechanical ventilation, reducing mortality.

## INTRODUCTION

Since infection by COVID-19 was first described, the severe respiratory syndrome associated with the disease has caused rapid increases in admissions to intensive care units (ICUs) and elevated mortality of a group of patients.^1^ During a pandemic, it is necessary to avoid saturation of health systems, both public and private, and in particular of ICUs. The principal relevant finding in the lungs is presence of platelet thrombi and fibrin in small arterial vessels, which fits perfectly with a clinical scenario of coagulopathy.^2^

Since there is no consensus-approved treatment in this situation and considering the possibility of thrombosis associated with infection by coronavirus in certain cases, recently-acquired experience and findings of still-embryonic scientific studies has shown that effective anticoagulation can prevent or reverse the prothrombotic state in some patients.^2^^,^^3^

## PROPOSAL

**Figure 1:**
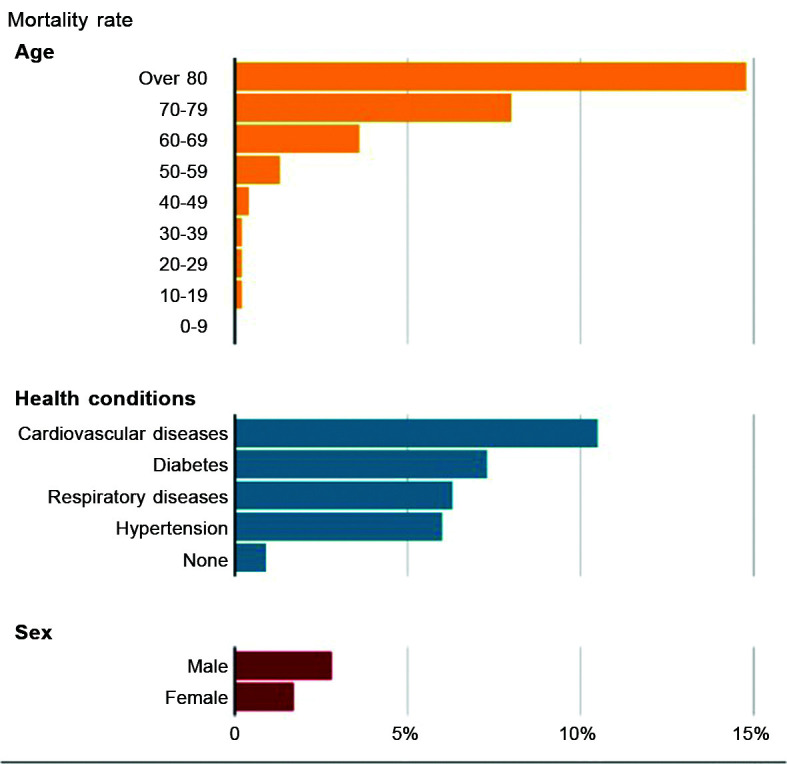
Mortality from COVID-19 varies by age and health status.^4^

We have observed that, coincidentally, the group of patients who respond poorly to the COVID-19 infection (Figure 1)^4^ and die are the same patients whose CHA_2_DS_2_-VASc scores indicate risk of stroke, transitory ischemic episode, peripheral emboli, and pulmonary thromboembolism (Table 1).^5^^,^^6^ According to this score, a patient is considered high risk if they score 2 points or more, intermediate risk if they score 1, and low risk if they do not have risk factors.^6^ Our proposal, therefore, is to add 1 point to the CHA_2_DS_2_-VASc score (Table 1) for patients who have COVID-19 and use the new score to indicate prophylactic anticoagulation for patients with a high risk of thrombosis according to the score, in phase 2 of the disease (Table 2). The objective is to prevent the patient’s condition from worsening because of thromboembolic problems, avoiding the need for ICU admission and mechanical ventilation.^7^

**Table 1:** Structure of the CHA_2_DS_2_-VASc score after addition of 1 point for COVID-19 (CHA_2_DS_2_-VASc-C19).

**CHA_2_DS_2_-VASc**	**Description**	**Points**
C	Heart failure	1
H	Hypertension	1
A_2_	Age (≥ 75 years)	2
D	Diabetes mellitus	1
S_2_	Prior TIA or stroke	2
V	Vascular disease (prior AMI, aortic plaque, peripheral arterial disease)	1
A	Age (65-74 years)	1
**C19**	Suspected or confirmed COVID-19	1

TIA = transient ischemic attack; AMI = acute myocardial infarction.

**Table 2:** Phases of COVID-19 infection and treatment.

**Phases**	**Clinical status**	**Treatment**
Phase 1	Flu-like respiratory infection	Avoid contagion, reduce symptoms, reduce viral load with medications in use
Phase 2 (see Table 1)	High risk of thrombosis	Prophylaxis, avoid intra pulmonary thrombosis, prophylactic anticoagulation
Phase 3	Critical patient in ICU	Full therapeutic anticoagulation

ICU = intensive care unit.

The idea is to proceed in a similar manner as with risk of thromboses and emboli according to the existing scores and initiate prophylaxis to attempt to avert occurrence of events that have contributed to the worsening clinical status of these patients.^1^^-^3 In this communication, the authors propose modifying the scoring of the CHA_2_DS_2_-VASc score and studying its validity, with the objective of reducing the number of critically patients who progress to phase 3.
