# Supplementary material for: Can we manage prophylactic therapy in COVID-19 patients to prevent severe illness complications?
Source: J Vasc Bras. 2020 May 20;19:e20200057. doi: 10.1590/1677-5449.200057 (PMC8202192; doi:10.1590/1677-5449.200057)
Supplement: Translation

# Translation: Podemos atuar preventivamente para evitar que os pacientes portadores de COVID-19 evoluam de forma mais grave?

**Authors:** Paulo Eduardo Ocke Reis, Marcos Cesar Braga Lima

**Affiliations:** 1 Universidade Federal Fluminense – UFF, Departamento de Cirurgia Geral e Especializada, Niterói, RJ, Brasil.

**Keywords:** COVID-19, coronavirus, anticoagulantes, trombose, prevenção, mortalidade hospitalar

## Abstract

Muitos pacientes com COVID-19 apresentam complicações tromboembólicas que acabam piorando seu prognóstico. Os autores propõem uma modificação no escore CHA_2_DS_2_-VASc, incluindo 1 ponto para COVID-19, para, desse modo, indicar profilaxia de eventos tromboembólicos antes do agravamento do quadro. As vantagens dessa modificação seriam evitar a piora do paciente por problemas tromboembólicos, bem como a necessidade de internação em unidade de tratamento intensivo e de ventilação mecânica, e diminuir a mortalidade.

## INTRODUÇÃO

Desde a primeira descrição da infecção por COVID-19, a síndrome respiratória grave associada à doença levou ao aumento rápido de admissões em unidades de tratamento intensivo (UTIs) e à alta mortalidade de um grupo de pacientes^1^. Em uma pandemia, é preciso evitar a saturação do sistema de saúde tanto público quanto privado, em especial das UTIs. O principal achado com relevância nos pulmões é a presença de trombos plaquetários e fibrina em pequenos vasos arteriais, enquadrando-se perfeitamente no contexto clínico da coagulopatia^2^.

Como não há um tratamento aprovado em consenso neste cenário e tendo em vista a possibilidade de trombose associada à infecção pelo coronavírus em determinados casos, a experiência adquirida recentemente e estudos científicos ainda embrionários têm mostrado que uma anticoagulação efetiva poderia prevenir ou reverter o estado pró-trombótico de alguns pacientes^2^^,^^3^.

## PROPOSTA

Observamos que, coincidentemente, o grupo de pacientes que evoluem mal da infecção por COVID-19 (Figura 1)^4^ e morrem são os mesmos pacientes do escore CHA_2_DS_2_-VASc com risco para acidente vascular cerebral, episódio isquêmico transitório, embolia periférica e tromboembolismo pulmonar (Tabela 1)^5^^,^^6^. Por esse escore, o paciente é considerado de alto risco se a pontuação for 2 ou mais, de risco intermediário se a pontuação for 1 e de baixo risco se não tiver fatores de risco^6^. A nossa proposta, portanto, é incluir 1 ponto adicional no escore CHA_2_DS_2_-VASc (Tabela 1) para pacientes portadores de COVID-19 e usar o novo escore para indicar a anticoagulação profilática nos pacientes com alto risco de trombose pelo escore, na fase 2 da doença (Tabela 2). Desse modo, busca-se evitar a piora do paciente por problemas tromboembólicos, bem como a necessidade de internação em UTI e de ventilação mecânica^7^.

**Figura 1:** A taxa de mortalidade por COVID-19 varia de acordo com a idade e a condição de saúde^4^.

**Tabela 1:** Como ficaria o escore CHA_2_DS_2_-VASc com o acréscimo de 1 ponto por COVID-19 (CHA_2_DS_2_-VASc-C19).

**CHA_2_DS_2_-VASc**	**Descrição**	**Pontos**
C	Insuficiência cardíaca	1
H	Hipertensão	1
A_2_	Idade (≥ 75 anos)	2
D	Diabetes mellitus	1
S_2_	AIT ou AVC prévio	2
V	Doença vascular (IAM prévio, placa aórtica, doença arterial periférica)	1
A	Idade (65-74 anos)	1
**C19**	Suspeita ou confirmação de COVID-19	1

AIT = ataque isquêmico transitório; AVC = acidente vascular cerebral; IAM = infarto agudo do miocárdio.

**Tabela 2:** Fases da infecção por COVID-19 e tratamento.

**Fases**	**Clínica**	**Tratamento**
Fase 1	Infecção respiratória gripal	Evitar contágio, diminuir sintomas, diminuir carga viral com as medicações que estão sendo usadas
Fase 2 (verificar Tabela 1)	Alto risco de trombose	Profilaxia, evitar trombose intrapulmonar, anticoagulação profilática
Fase 3	Paciente grave em UTI	Anticoagulação plena terapêutica

UTI = unidade de tratamento intensivo.

A ideia é agir de forma similar ao risco de tromboses e embolias pelos escores conhecidos e iniciar a profilaxia para tentar evitar a ocorrência do que tem contribuído para o agravamento do quadro clínico desses pacientes^1^^-^3. Através deste artigo, os autores propõem uma modificação pontual na escore CHA_2_DS_2_-VASc para estudo de sua validação, com o objetivo de diminuir o número de doentes críticos que chegam à fase 3.
